# High‐Resolution Mechanoluminescent Haptic Sensor via Dual‐Functional Chromatic Filtration by a Conjugated Polymer Shell

**DOI:** 10.1002/adma.202508917

**Published:** 2025-08-14

**Authors:** Hong In Jeong, So Eun Choi, Xian Wei Chua, Nam Woo Kim, Eleni Pyrilli, Hyosun Lee, Dong‐Won Kang, Bo Ram Lee, Samuel D. Stranks, Jongho Kim, Sujoy Bandyopadhyay, Hyosung Choi

**Affiliations:** ^1^ Department of Chemical Engineering and Biotechnology University of Cambridge Cambridge CB3 0AS UK; ^2^ Department of Chemistry Research Institute for Natural Sciences Research Institute for Convergence of Basic Science Hanyang University Seoul 04763 Republic of Korea; ^3^ Department of Textile System Engineering Kyungpook National University Daegu 41566 Republic of Korea; ^4^ School of Energy Systems Engineering Chung‐Ang‐University Seoul 04763 Republic of Korea; ^5^ School of Advanced Materials Science and Engineering Sungkyunkwan University Suwon 16419 Republic of Korea

**Keywords:** chromatic filtration of conjugated polymer, hyperspectral photoluminescence microscopy, mechanoluminescence platform, mechanoluminescence, mechanoluminescent haptic sensor

## Abstract

Mechanoluminescence materials have emerged as promising candidates for haptic interface sensors due to their mechanically driven luminescent property. However, the inherently broad emission spectra of most mechanoluminescence materials hinder sharp signal generation and high spectral resolution. Here, a chromatic filtration strategy is suggested employing poly(9,9‐dioctylfluorene‐alt‐benzothiadiazole) (F8BT) as a conjugated polymer shell on copper‐doped zinc sulfide to achieve a highly refined and intensified signal. By selectively suppressing spectral components below 490 nm, the F8BT shell effectively narrows the emission bandwidth, yielding a full width at half maximum (FWHM) of 55 nm during the mechanoluminescence process. In particular, F8BT shell efficiently mitigates signal intensity loss, which can be ascribed to the chromatic filtration ability, through mechanoluminescence photon recycling performance. This dual functionality significantly reduces spectral noise in the blue region with high intensity, enhancing the resolution in actual powerless haptic controllers. The novel approach establishes a scalable framework for high‐resolution mechanoluminescence platforms, providing a versatile pathway toward next‐generation, power‐free stress‐sensing applications with unprecedented spectral precision and optical fidelity.

## Graphical abstract

A high‐resolution mechanoluminescence (ML) platform is achieved through chromatic filtration strategy using a conjugated polymer shell (F8BT) on copper‐doped zinc sulfide microparticles. This shell narrows the emission bandwidth, selectively filtering out blue noise, while simultaneously boosting intensity via ML photon recycling process. The dual action significantly enhances signal clarity and stability, enabling precise multi‐signal differentiation in haptic sensors and advanced stress‐sensing technologies.

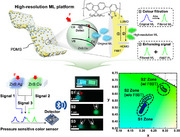

## 1 Introduction

The advancement of next‐generation sensing technologies, such as bite‐controlled user interface,^[^ ^1^ ^]^ healthcare motion monitoring,^[^ ^2^, ^3^, ^4^ ^]^ and piconewton sensing,^[^ ^5^ ^]^ has opened new frontiers in mechanoluminescence (ML) materials, which exhibit self‐powered and recoverable luminescence properties. In particular, copper‐doped zinc sulfide (ZnS:Cu) has emerged as one of the most promissing candidates due to its superior performance^[^ ^6^, ^7^ ^]^ and compatibility with diverse polymer matrices.^[^ ^4^, ^8^, ^9^, ^10^ ^]^ However, one of the major limitations of ML materials, particularly in realistic sensing applications,^[^ ^11^ ^]^ is their inherently broad emission spectrum,^[^ ^7^, ^12^ ^]^ which significantly degrades signal resolution and introduces spectral noise.

The broad emission profile of ZnS:Cu often originates from the phosphorescence process associated with Cu dopant states,^[^ ^13^ ^]^ which are essential for the ML effect but result in overlapping spectral components. This poses a critical challenge for mechanical motion tracking applications requiring high spectral resolution.^[^ ^14^, ^15^ ^]^ Additionally, some machine learning‐assisted force tracking controller systems requiring multiple signal types may suffer from inaccuracies due to low signal resolution and significant spectral overlap.^[^ ^1^ ^]^

For example, emission overlap caused by low resolution significantly hinders the ability to achieve precise signal control and clear visual output in mechano‐to‐luminescence processing applications, including pencil‐pressure sensing,^[^ ^16^, ^17^ ^]^ self‐powered displays,^[^ ^7^, ^18^ ^]^ and healthcare motion controlling systems.^[^ ^3^, ^19^ ^]^ This not only complicates multiple color signal processing but also reduces the overall effectiveness of these systems in accurately translating mechanical stimuli into readable data. Fundamentally, the ML signal of doped ZnS exhibits different colors and emission intensities depending on the specific dopants, such as Ag, Cu, and Mn.^[^ ^20^ ^]^ This implies that in multi‐signal processing systems utilizing two or more different materials, one of the signals can be easily overshadowed by another with much higher intensity, especially under varying pressure levels.^[^ ^1^, ^20^, ^21^ ^]^ Therefore, enabling high‐resolution signal differentiation and achieving clear spectral separation is a crucial breakthrough in sensing technologies that leverage ML materials.

While some research has explored color manipulation of ML materials by means of the reabsorption and reemission effects of additional high‐color‐purity emitters such as quantum dots^[^ ^18^, ^22^, ^23^ ^]^ and polymer‐based materials,^[^ ^24^ ^]^ most studies have struggled to avoid significant degradation of ML signal intensity due to the partial suppression of the original ML spectrum. The study of chromatic filtration and signal improvement by using shell structures with highly absorptive and luminescent polymers remains largely unexplored to date.

Here, we present a novel chromatic filtration strategy employing a conjugated polymer shell of poly(9,9‐dioctylfluorene‐alt‐benzothiadiazole) (F8BT) on ZnS:Cu microparticles to achieve a high‐resolution ML platform. By considering the superior absorption of F8BT in the blue region below 490 nm, which constitutes significant noise in the green signal, a portion of the ZnS:Cu emission spectrum is selectively filtered in the short wavelength range, effectively narrowing the full width at half maximum (FWHM) of the emitted signal to 55 nm under mechanical stimulus. In particular, we reveal that the F8BT shell compensates for signal intensity loss caused by the chromatic filtration effect through its outstanding ML photon recycling performance. By using our strategy, we demonstrated signal separation with high ML intensity in a multi‐button press‐tracking sensor utilizing typical doped ZnS with severe spectral overlap between blue and green signals. Our findings provide new perspectives on the material design principles for next‐generation ML‐based sensors, opening possibilities for advanced stress‐sensing technologies with high detection accuracy and minimal spectral interference.

## 2 Results and Discussion

### 2.1 Structural and Morphological Property of High‐Resolution ML Platform

**Figure 1:**
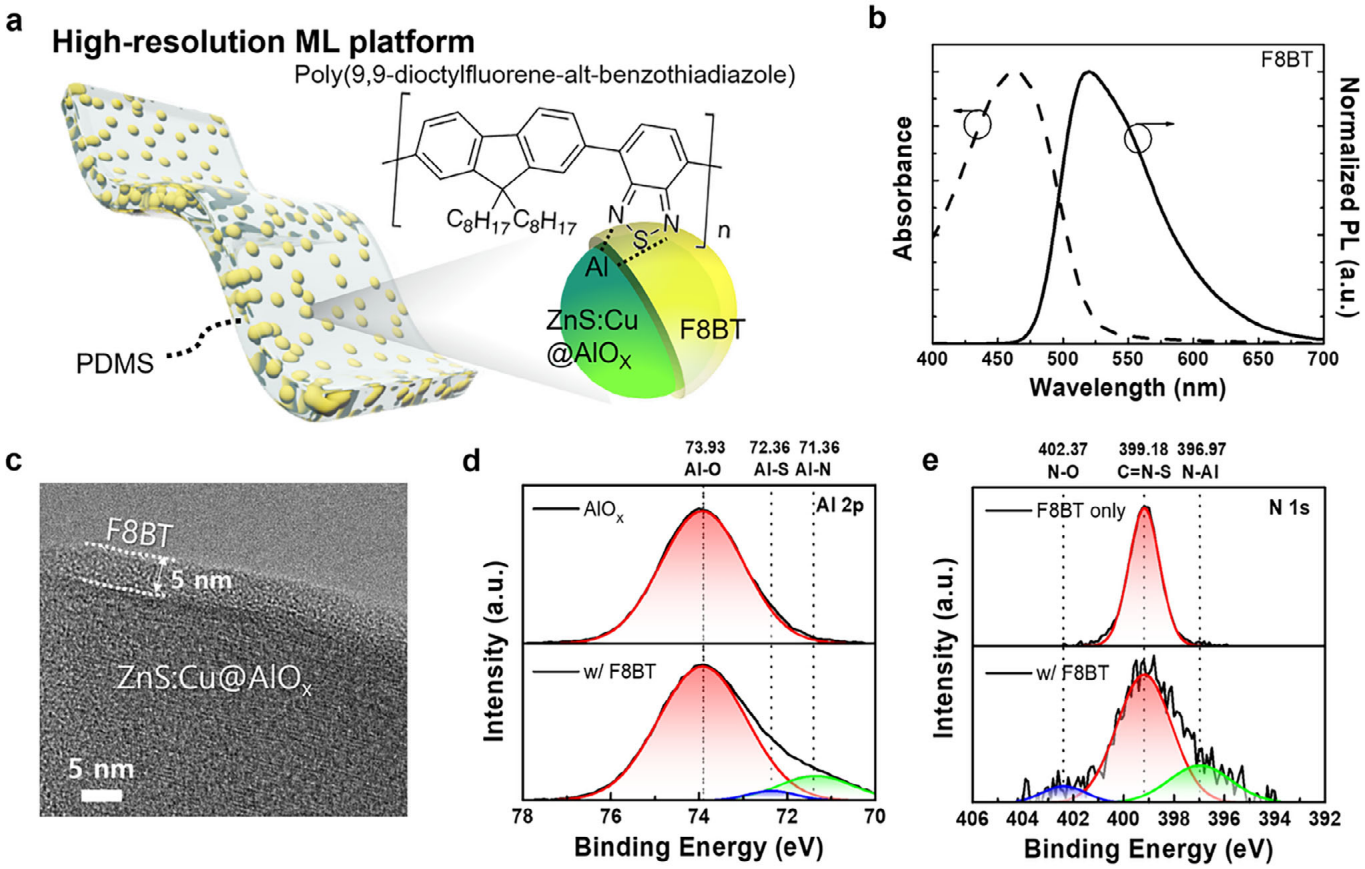
a) Schematic structure of a high‐resolution ML platform consisting ZnS:Cu@F8BT microparticles embedded in the PDMS matrix. b) Absorbance and PL spectrum of an F8BT polymer thin film. c) TEM image exhibiting the thickness of the F8BT shell on the ZnS:Cu single particle. XPS spectra of d) the Al 2p core level, comparing uncoated AlO_X_ and AlO_X_–F8BT, and e) the N 1s core level of neat F8BT and AlO_X_–F8BT, supporting the formation of interfacial bonding between an F8BT and ZnS:Cu.

We first prepared a typical ML platform consisting of alumina (AlO_X_)‐coated ZnS:Cu microparticles (denoted as ZnS:Cu) embedded in a polydimethylsiloxane (PDMS) matrix (**Figure** **1**). Since ZnS:Cu is well known to exhibit green ML emission with a significant portion in the blue spectral region (450–490 nm) (Figure S1, Supporting Information), we selected an F8BT‐conjugated polymer as a chromatic filter due to its high absorption in the blue region (inset in Figure 1a). To confirm the possibility of chromatic filtration range and reemission, we characterized the absorption and PL properties of an F8BT thin film (Figure 1b). Strong absorption was observed in the blue region of wavelengths below 490 nm and manifested as high‐intensity PL at ≈510 nm. This means that F8BT exhibits an appropriate overlap between its absorption range and the blue emission region of ZnS: Cu, which could be a significant noise signal, making it suitable for use as a chromatic filter.

Based on this F8BT polymer, we preferentially established a solution‐based shell coating process to fabricate ZnS:Cu particles with an F8BT shell (ZnS:Cu@F8BT). By using transmission electron microscopy (TEM), we first demonstrated the shell‐forming ability of F8BT by comparing the ZnS:Cu surface before and after coating with a high‐concentration F8BT precursor solution (Figure S2a–d, Supporting Information). As shown in Figure S2a (Supporting Information) and b, ZnS:Cu microparticles without F8BT coating show no discernible shell formation on their surfaces. In contrast, microparticles coated with F8BT at 5 mg mL^−1^ exhibited a clearly defined and uniform F8BT shell across all surfaces of the imaged particles (Figure S2c,d, Supporting Information). Based on this observation, we optimized the F8BT shell thickness on ZnS:Cu microparticles by governing the F8BT concentration in the precursor solution. As the F8BT concentration increased from 1 to 6 mg mL^−1^, the thickness of the F8BT shell increased proportionally from 2 to 5 nm (Figure S2e–h, Supporting Information). Beyond 4 mg mL^−1^, notably, no significant further increase in shell thickness was observed, suggesting a saturation behavior (Figure S2i, Supporting Information), and therefore 5 mg mL^−1^ was selected as the optimized concentration for shell formation (Figure 1c). In all conditions with different concentration of F8BT, although each shell formed on the surface exhibited some degree of non‐uniformity due to the solution process, the average thickness was found to be well maintained. Across all TEM images depending on the different F8BT concentrations, the shells exhibited slight non‐uniformity due to the solution process, but their average thickness remained well maintained. Additionally, to further prove that such slight thickness non‐uniformity does not affect the quality of the entire particle surface coating, we accessed scanning electron microscopy with energy‐dispersive X‐ray spectroscopy (SEM/EDX) mapping according to the different F8BT concentration (Figure S3, Supporting Information). As the F8BT concentration increased from 0 to 5 mg mL^−1^, SEM/EDX mapping revealed a homogeneous distribution of the C signal across the entire particle surface (Figure S3a–c, Supporting Information), and the C atomic percentage correspondingly increased from 0% to 42.3% (Table S1, Supporting Information), further confirming the formation of a uniform F8BT shell.

To confirm the stable adhesion of the F8BT layer for mechanical stability during the ML operation, we utilized X‐ray photoelectron spectroscopy (XPS) to probe between the F8BT and AlO_X_ surface of ZnS:Cu (Figure 1d,e). The Al 2p region was compared between uncoated ZnS:Cu and F8BT‐coated ZnS:Cu surfaces. We observed the presence of new Al–S and Al–N bonding environments, suggesting that the F8BT shell modifies the surface chemistry of AlO_X_ (Figure 1d). For the N 1s core‐level spectra of neat F8BT and AlOx–F8BT, clear peaks corresponding to N–Al and N–O interactions can be confirmed (Figure 1e). Considering the functional group on the F8BT and AlO_X_ on ZnS:Cu surface (inset in Figure 1a), these spectral features confirm the successful formation of effective interaction between the AlO_X_ surface and the nitrogen‐containing F8BT polymer backbone.

### 2.2 High‐Resolution ML Performance by Chromatic Filtration of F8BT Shell

**Figure 2:**
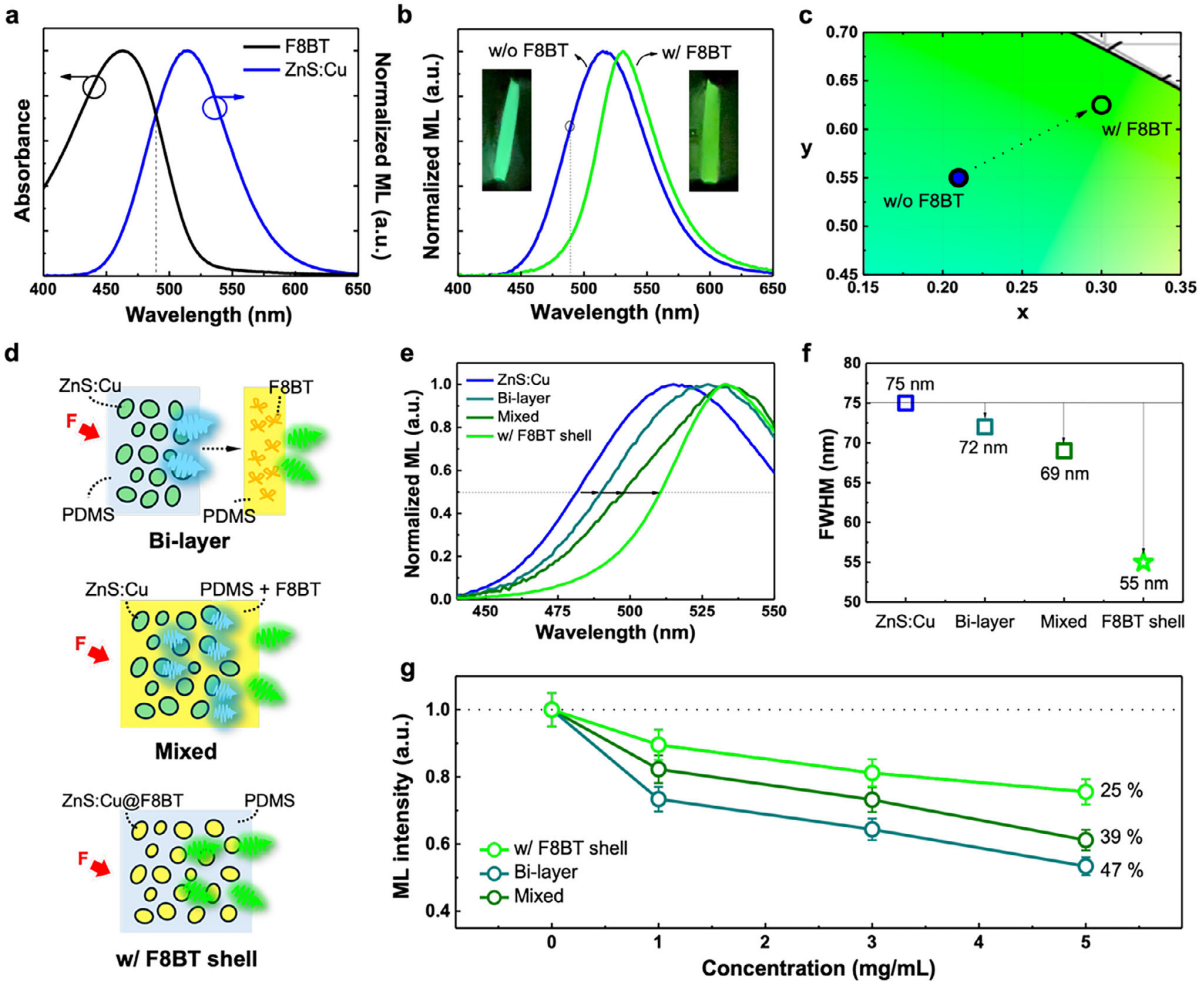
a) Absorption spectrum of the F8BT polymer and ML spectrum of ZnS:Cu exhibiting the spectral overlap in the blue region below 490 nm for the potential chromatic filtration. b) ML spectra and c) CIE color coordinates of ZnS:Cu with and without F8BT shell. Insets in (b) show the real ML images corresponding to each spectrum. d) Sample design for evaluating the chromatic filtration capability based on the positioning of the F8BT polymer, featuring bi‐layer, mixed, and w/ F8BT shell configurations. e) ML spectra and f) FWHM of ZnS:Cu as a function of different F8BT positions in the ML platform, corresponding to (d). g) Relative ML intensity of different samples in (d) as a function of F8BT concentration (*n* = 5, error bars represent ±8%).

To verify the ability of chromatic filtration of F8BT for high‐resolution ML with refined spectral characteristics, we investigated the ML performance under various environments of F8BT. The chromatic filtration potential of F8BT was first assessed by comparing its absorption spectrum with the intrinsic ML spectrum of ZnS:Cu (**Figure** **2**a). Given the significant overlap between the absorption profile of F8BT and ML emission of ZnS:Cu in the blue region ≈490 nm, we explored the possibility of selectively attenuating these shorter‐wavelength components during the ML behavior. Indeed, the F8BT shell effectively filtered out ML emission below 490 nm due to its strong absorption properties, thereby manifesting a narrow spectrum for the entire ML platform (Figure 2b). This effect is further validated by the Commision Internationale de I'Eclairage (CIE) color coordinates (Figure 2c), where the ZnS:Cu@F8BT exhibits a distinct shift toward the pure green region at (0.30, 0.65), effectively suppressing blue emission and reinforcing the efficiency of the chromatic filtration ability toward high‐resolution signal (inset in Figure 2b). This spectral interaction between F8BT and ZnS:Cu in ML operation provides a fundamental possibility for achieving a more defined high‐resolution ML signal profile.

To further elucidate the chromatic filtration impact of F8BT in the ML platform, three distinct structured samples were designed (Figure 2d). The bi‐layer sample consisted of a ZnS:Cu‐PDMS layer with an additional F8BT‐PDMS film on top (top of Figure 2d). The mixed sample contained ZnS:Cu microparticles dispersed in a PDMS matrix blended with F8BT (middle of Figure 2d). The F8BT shell sample consisted of ZnS:Cu microparticles each coated with an F8BT shell before being embedded in the PDMS matrix (bottom of Figure 2d). Based on these different sample structures, we evaluated the chromatic filtration ability through the ML spectrum focusing on the blue region (Figure 2e). This comparison revealed that the ZnS:Cu@F8BT sample exhibited the most effective chromatic filtration, followed by the mixed sample and the bi‐layer sample. This trend was further quantified through FWHM analysis (Figure 2f), where the ZnS:Cu@F8BT sample achieved the narrowest bandwidth of 55 nm, significantly reduced compared with 69 nm for the mixed sample and 72 nm for the bi‐layer sample. By contrast, uncoated ZnS:Cu exhibited a substantially broader emission with an FWHM of 75 nm, highlighting the superior efficiency of the F8BT shell in selectively suppressing undesired spectral components.

Building on this result, proving the feasibility and advantages of the ZnS:Cu@F8BT sample is crucial for rationalizing the effect of chromatic filtration. Since ML occurs in a stretchable environment, we examined the absorption and transmittance of the PDMS matrix with varying F8BT concentrations, as used in mixed F8BT‐PDMS samples, under stretching conditions (Figure S4, Supporting Information). The F8BT‐PDMS sample exhibited an increase in absorption with higher F8BT concentrations under static conditions (Figure S4a, Supporting Information). However, under stretching, a significant increase in transmittance was observed, indicating that the matrix allows more light to pass through when mechanically deformed (Figure S4b, Supporting Information). This suggests that while F8BT effectively absorbs shorter‐wavelength emission in a static state, its filtration efficiency may vary under mechanical strain, potentially impacting the consistency of chromatic filtration. In the bi‐layer sample, the F8BT‐PDMS layer mostly attenuated the emitted ML light, ensuring that the chromatic filtration effect remains consistent across the entire emission pathway. By contrast, the mixed sample exhibited relatively low absorption efficiency due to the random surface exposure of ZnS:Cu microparticles (Figure S5, Supporting Information, EM), which creates pathways for unfiltered emission. Consequently, the particle‐by‐particle F8BT shell coating proved to be the most effective approach for ML processes, ensuring superior absorption and acting as an optimal chromatic filter. This structure not only maximizes spectral refinement but also maintains consistent filtration performance regardless of mechanical deformation.

The signal intensity is also a crucial factor in determining sensing performance with high‐resolution. Since chromatic filtration inherently involves absorption of a certain spectral range, a reduction in ML intensity is usually expected. To quantify this effect, the relative ML spectra of each sample was evaluated as a function of F8BT concentration ranging from 0 to 5 mg mL^−1^ (Figure S7, Supporting Information). As shown in Figures S7a,c,e (Supporting Information), all samples displayed a linear decrease in ML intensity with increasing F8BT concentration, which is ascribed to the high absorption ability of F8BT. As we discussed in Figure 2e, the ZnS:Cu@F8BT sample manifested consistently high chromatic filtration capability at all concentrations compared with another samples (Figure S7b,d, f, Supporting Information) and maintained higher ML performance across all concentrations. The relative ML intensities of each sample at different concentrations are shown in Figure 2g. In particular, at the F8BT concentration of 5 mg mL^−1^, which provides the most pronounced chromatic filtration, the ZnS:Cu@F8BT structure exhibited the lowest loss in ML intensity, with only a 25% reduction, whereas the mixed and bilayer samples showed significantly higher losses of 39% and 47%, respectively (relative spectra are shown in Figure S8a, Supporting Information). Additionally, these samples exhibited a linear trend across a wide tensile strain range of 30–60%, and unlike the bi‐layer and mixed samples, which fractured at over 50% strain condition due to the direct embedding of F8BT into PDMS, ZnS:Cu@F8BT maintained the highest ML performance throughout the entire strain range (Figure S8b, Supporting Information).

Given the highest absorption rate of the ZnS:Cu@F8BT sample, which directly correlates with its superior chromatic filtration capability, it would be expected to experience the greatest intensity loss due to absorption of a significant portion of the emitted light. However, the fact that this sample exhibits the least intensity loss is unexpected. Despite the absorption caused by chromatic filtration and the absence of any noticeable surface modification effect (Figure S9 and Note S1, Supporting Information), which could theoretically enhance ML performance through interfacial triboelectric effects (Figure S10, Supporting Information),^[^ ^2^, ^25^ ^]^ the exceptional intensity stability of the ZnS:Cu@F8BT sample suggests that the F8BT shell efficiently compensates for the ML effect. The minimal intensity loss observed in this configuration indicates a possible ML‐induced reabsorption and secondary emission mechanism, which is further explored in the following section.

### 2.3 Chromatic Filtration Mechanism of the High‐Resolution ML Platform

To investigate how the F8BT shell enables both high‐resolution emission and minimal intensity loss, we first compared the PL and ML behaviors of the two primary constituents: ZnS:Cu and the F8BT shell. Specifically, we examined the PL and ML spectra of bare ZnS:Cu (Figure S11a, Supporting Information) as a reference for subsequent spectral modifications. Under PL excitation, ZnS:Cu shows a dominant blue emission peak at 450 nm, whereas its ML emission is centered at 510 nm with a broader Gaussian profile. A similar spectral difference appears in the ZnS:Cu@F8BT system (Figure S11b, Supporting Information). For the PL, we find that the overall spectrum includes not only 450 nm emission corresponding to a key peak position of ZnS:Cu but also strong 510 nm emission ascribed to the F8BT shell. By contrast, in the case of ML, a single intense peak at ≈510 nm dominates, implying that the ZnS:Cu ML may be exciting the F8BT shell, leading to a secondary emission. In an effort to unveil this possibility for ML photon recycling effect in F8BT shell, we accessed photoluminescence excitation (PLE) with a F8BT‐PDMS matrix over an excitation range of 440 to 500 nm, which is the overlap from the chromatic filtration range (Figure S11c, Supporting Information). A noticeable intensity increase around the filtration range of 450–500 nm, which aligns well with the ML of ZnS:Cu, is observed. This possibility of photon recycling process in the F8BT shell by ML was proved by employing the F8BT‐PDMS sample, as described in the previous section (Figure 2d). The reabsorption and photon recycling process associated with F8BT is exhibited by the ML from ZnS:Cu (Figure S12, Supporting Information). Moreover, raising the F8BT content in the film proportionally amplified the F8BT emission, reinforcing the idea that ZnS:Cu‐generated photons are absorbed and re‐emitted by the shell. These observations strongly support the hypothesis that ML‐generated photons from ZnS:Cu excite F8BT, leading to secondary emission that not only enhances spectral refinement but also compensates for intensity loss. Hence, the F8BT shell emerges as an active participant in the overall emission process, rather than merely serving as a passive filter at 510 nm.

**Figure 3:**
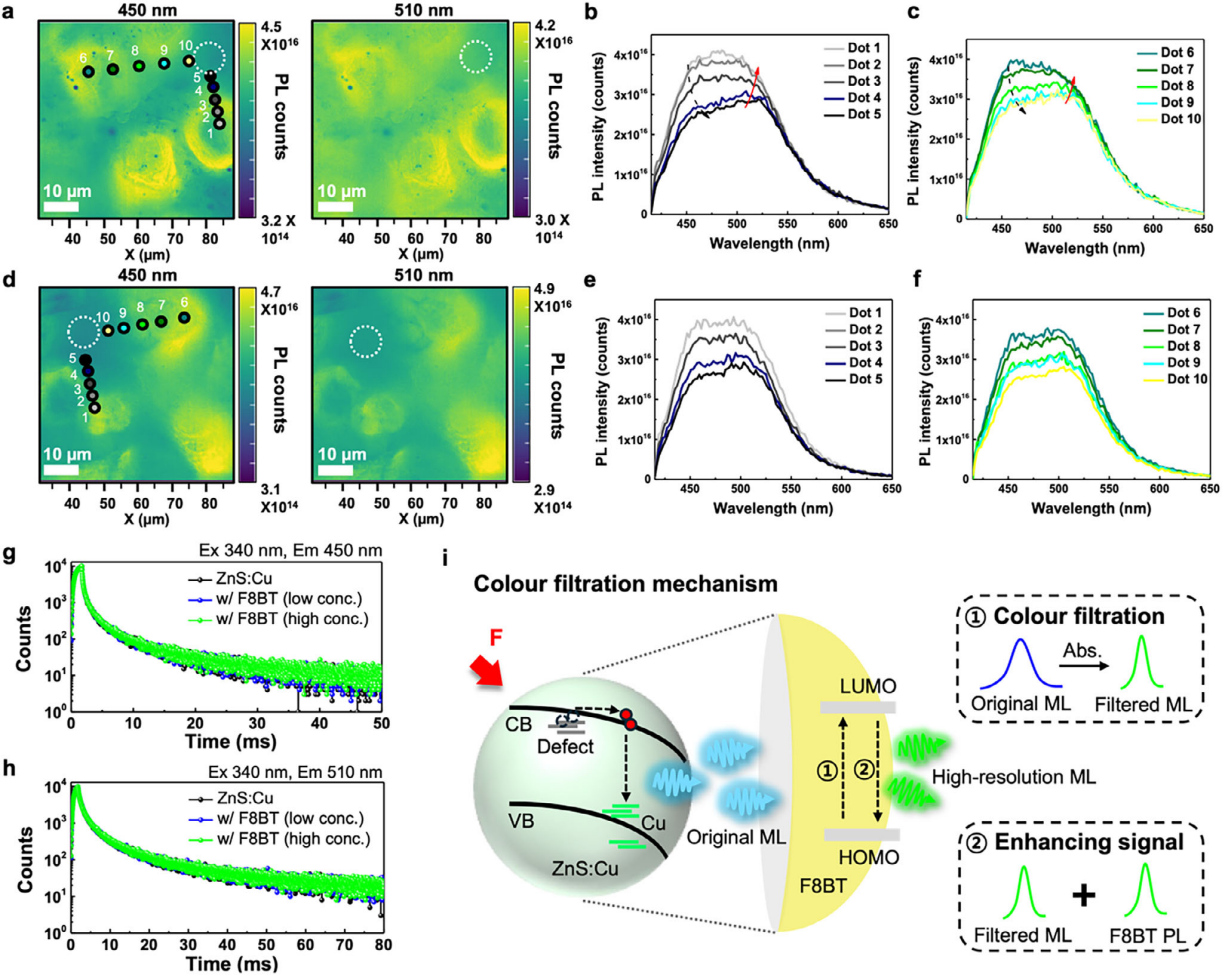
Hyperspectral maps of a) ZnS:Cu in PDMS with F8BT, d) ZnS:Cu@F8BT in PDMS samples, and spectra in point region of maps (b,c) dots of 1–10 in (a) and e,f) dots of 1–10 in (d)) as a function of 450 and 510 nm emission with excitation wavelength at 405 nm and intensity of 36 mW cm^−2^. Time‐resolved PL of ZnS:Cu at g) 450 nm and h) 510 nm emission components, depending on the F8BT concentration. i) Chromatic filtration mechanism in the high‐resolution ML platform through both the chromatic filtration effect and ML photon recycling effect by the F8BT shell.

Proving the spectral interactions on a microscale landscape is crucial for clarifying how ZnS:Cu and F8BT components interact locally. To gain further insight into the ML photon recycling process, we performed hyperspectral photoluminescence (PL) microscopy on two sample configurations. We first analyzed a mixed sample, composed of bare ZnS:Cu microparticles dispersed in an F8BT‐PDMS matrix, and obtained PL intensity maps at emission wavelengths of 450 and 510 nm (**Figure** **3**a). We then selected several points of interest (dots 1–10 in Figure 3a) moving outward from the ZnS:Cu particle into the surrounding F8BT‐PDMS region. Their corresponding spectra are shown in Figure 3b (dots 1–5) and Figure 3c (dots 6–10). Notably, the 450 nm emission gradually weakens as the dot number increases toward the matrix, whereas the 510 nm peak gains intensity, indicating that ZnS:Cu‐originated photons excite F8BT in the polymer matrix. Additionally, the dotted circular area highlighted in the PL maps shows negligible emission at 450 nm but strong emission at 510 nm, supporting the hypothesis that F8BT undergoes secondary emission after reabsorbing the ZnS:Cu photons.

By contrast, the ZnS:Cu@F8BT sample embedded in PDMS shows no pronounced difference in emission intensity between the particle and the surrounding matrix at either 450 or 510 nm (see dotted circles in Figure 3d). This underscores both the positional effect of the F8BT shell and its photon recycling contribution, reaffirming how the shell ensures uniform emission across the core–shell interface. Notably, for the selected points of interest (dots 1–10 in Figure 3d) moving from the particle outward, each measured point exhibits a relatively consistent spectral shape (Figure 3e,f). Although the overall PL intensity may vary slightly, the relative balance between the 450 and 510 nm components remains similar at all points. Such uniformity implies that once ZnS:Cu is encapsulated by the F8BT shell, the ML photon recycling process takes place uniformly around each microparticle—largely independent of the external polymer matrix.

To quantify these observations, we extracted horizontal line‐scan profiles for selected rows in the PL maps (Figure S13, Supporting Information). In the mixed sample (Figure 3a), the 450 nm signal spikes at the ZnS:Cu particle region but drops off in the polymer. By contrast, in the ZnS:Cu@F8BT sample (Figure 3d), the relative intensities of 450 and 510 nm remain more consistent across the entire line, underscoring the homogeneous emission behavior provided by the F8BT shell. We further examined a bi‐layer sample comprising a top F8BT‐PDMS film in contact with ZnS:Cu (Figure S14a,b, Supporting Information). Here, the hyperspectral map revealed almost no emission at 450 nm but a strong signal at 510 nm for all measured points (dots 1–5), as seen in Figure S14c,d (Supporting Information). This result confirms that F8BT in the upper layer efficiently reabsorbs and re‐emits ZnS:Cu light, reinforcing its active role in color filtration and secondary PL emission. Taken collectively, these hyperspectral analyses demonstrate that the F8BT shell (or layer) not only suppresses the shorter‐wavelength ZnS:Cu emission but also re‐emits at 510 nm in a stable, uniform manner, providing clear evidence of the dual‐action chromatic filtration and ML photon recycling mechanism outlined in our study.

To substantiate this mechanism, we conducted time‐resolved PL (TRPL) measurements to rule out the possibility of charge transfer (CT) and energy transfer (ET) processes at 450 nm (ZnS host) and 510 nm (dopant‐related) emission wavelengths under varying F8BT concentrations (Figure 3g,h). The average lifetime (τ_avg_) of the TRPL decay curves was determined by fitting the experimental data to a multi‐exponential decay function:

(1) $$I\;\left(t\right)=A_{1}e^{-1/\tau_{1}}+A_{2}e^{-1/\tau_{2}}+A_{3}e^{-1/\tau_{3}}$$

where A_i_ and τ_i_ represent the amplitude and lifetime of each decay component, respectively. The amplitude‐weighted average lifetime was then calculated using:

(2) $$\tau_{avg}=\frac{\sum A_{i}{\tau_{i}}^{2}}{\sum A_{i}\tau_{i}}$$

This approach accounts for contributions from both fast and slow decay components. Fitting quality was evaluated using the chi‐squared (χ^2^) statistic and residual analysis to ensure the reliability of the multi‐exponential fitting. The resulting average lifetimes at both 450 and 510 nm (Table S2, Supporting Information) appeared similar across all F8BT concentrations (control, low, medium, and high), with average values of ≈3.52 ms at 450 nm and 6.39 ms at 510 nm. This indicates that F8BT exerts only a negligible or very subtle influence on the ZnS:Cu kinetics. This finding rules out direct electronic interactions between ZnS:Cu and F8BT, confirming that the observed PL enhancement arises solely from ML reabsorption and subsequent reemission by F8BT.

Based on these results, we propose a comprehensive chromatic filtration and ML signal enhancement mechanism (Figure 3i). When mechanical force is applied, ZnS:Cu generates ML light, which undergoes two concurrent steps within the F8BT shell. First, the shorter‐wavelength region (blue) is selectively absorbed, effectively refining the ML emission. Second, the remaining ML photons excite F8BT, triggering additional emission at ≈510 nm. This secondary emission compensates for the intensity loss otherwise caused by color filtration, allowing the ML platform to maintain a spectrally refined, high‐intensity output.

Hence, rather than acting solely as a passive optical filter, the F8BT shell performs two key functions, enhancing spectral resolution and minimizing intensity loss via ML photon recycling effect. This multi‐functional mechanism, in which F8BT simultaneously improves spectral purity and stabilizes ML intensity, paves the way for high‐performance ML‐haptic interface sensors with superior optical precision and signal fidelity.

### 2.4 Press‐Sensitive Chromatic Tracking System Based on the High‐Resolution ML Platform

**Figure 4:**
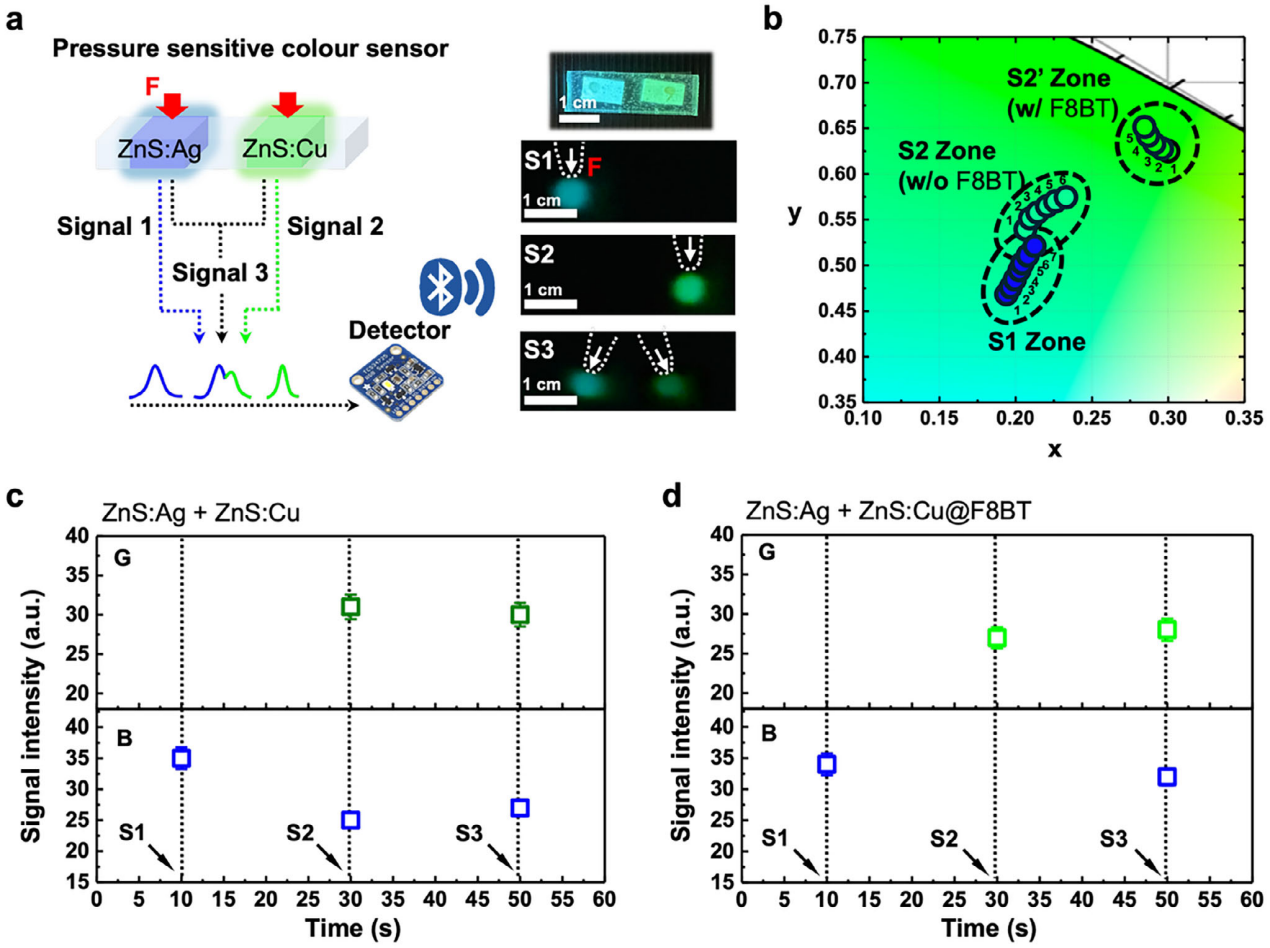
a) Schematic of the press‐sensitive color sensor with two‐buttons system. ZnS:Ag emits the blue ML signal, and the other ZnS:Cu emits the green signal. Signal 1 (S1) corresponds to pressing the blue ML button, Signal 2 (S2) to pressing the green ML button, and Signal 3 (S3) to pressing both simultaneously. b) CIE color coordinates of the emitted ML signals, where the S1 zone represents the blue ML emission, the S2 zone corresponds to pure ZnS:Cu, and the S2’ zone indicates the F8BT modified ZnS:Cu signal. c,d) Signal separation capability of the press‐sensitive color sensor under sequential pressing conditions, showing the intensity variations over time (*n* = 5, error bars represent ±7%).

Based on our developed high‐resolution ML platform through chromatic filtration strategy from the F8BT shell, we implemented a haptic button system to assess the signal differentiation capability (**Figure** **4**a). The press‐sensitive color tracking sensor, which consists of two independent buttons designed to generate distinct blue (ZnS:Ag) and green (ZnS:Cu) ML signals is prepared. Considering the difficulty in separating the signals from ZnS:Ag and ZnS:Cu in this ML haptic sensor systems, we defined the severely overlapping blue spectral region as the noise region for ZnS:Cu (Figure S15a, Supporting Information) and then investigated ML signal as a spectrum from two candidates. While the original ZnS:Cu exhibits significant overlap with ZnS:Ag, effective suppression of spectral crosstalk is observed for ZnS:Cu@F8BT, whose emission is almost entirely shifted out of the noise region. To further quantify this effect, we calculated signal‐to‐noise ratio (SNR) calculation of ZnS:Cu with and without the F8BT shell using the following formula: SNR = *I*_Green region_/*I*_Blue region_, where *I*_Green peak_ is the maximum intensity in the green spectral region (500–550 nm) and *I*_Blue region_ is the integrated intensity of the overlapping blue spectral region (400–490 nm). As shown in Figure S15b (Supporting Information), the SNR of the ML signal with the F8BT shell was significantly enhanced compared to the original sample without the shell (0.06 to 0.52). This high SNR clearly reveals the effectiveness of the F8BT shell in suppressing spectral overlap and enhancing signal clarity for ML haptic system.

To prove this result in haptic button system, two key signals are defined as a blue emission of ZnS:Ag (Signal 1, S1) and green emission of ZnS:Cu (Signal 2, S2). Additionally, when both buttons are pressed simultaneously, a combined signal (Signal 3, S3) is defined. The images in Figure 4a illustrate the individual ML signals corresponding to each button press, providing a visual confirmation of the signal separation capability. We then evaluate their CIE color coordinates to distinguish between the signal zones (Figure 4b), and the coordinates corresponding to the dot numbers indicated in each signal zone are listed in Table S3 (Supporting Information). The blue signal (S1) is clearly defined in the S1 zone, representing the ZnS:Ag emission with CIE color coordinates clustered ≈x  =  0.200 ± 0.007, y  =  0.495 ± 0.018. However, the green signal (S2) from conventional ZnS:Cu exhibits substantial spectral overlap with the blue region, leading to poor signal differentiation. This is evident from the S2 zone, where the average coordinates (x  =  0.217 ± 0.010, y  =  0.556 ± 0.020) show only a modest shift from the S1 zone (Δx  =  +0.017, Δy  =  +0.061), indicating that the green emission remains indistinct due to blue contamination. By contrast, the ZnS:Cu@F8BT ML platform effectively eliminates this overlap. In the S2′ zone, the F8BT‐enhanced green signal is clearly separated from the blue region, with average coordinates of x  =  0.291 ± 0.006, y  =  0.635 ± 0.010. This represents a substantial displacement relative to the S1 zone (Δx  =  +0.091, Δy  =  +0.140) and even relative to the S2 zone (Δx  =  +0.074, Δy  =  +0.079), highlighting the effective suppression of spectral crosstalk. This distinct shift underscores the superior spectral resolution achieved through our chromatic filtration approach, ensuring precise differentiation of color signals in a haptic interface.

To further substantiate the high‐resolution signal differentiation capability of our platform, we conducted real‐time signal intensity measurements under sequential button pressing conditions (Figure 4c,d). When the blue button (S1) was pressed, a well‐defined blue signal was detected (Figure 4c). However, when the S2 button (ZnS:Cu) was pressed, both blue and green signals were simultaneously activated, demonstrating significant spectral crosstalk and poor resolution in distinguishing independent signals.

By contrast, in the case of ZnS:Cu@F8BT, a clear separation between the blue and green signals was observed (Figure 4d). The S2 button generated a well‐defined green signal without any interference noise from the blue region, confirming that our high‐resolution ML platform successfully eliminates spectral overlap and enables precise multi‐signal differentiation. This enhanced resolution directly addresses the limitations of conventional ML‐based systems, offering a robust solution for haptic sensors, multi‐channel input systems, and next‐generation human‐machine control interfaces.

## 3 Conclusion

In this study, we introduced a chromatic filtration strategy utilizing an F8BT polymer shell on ZnS:Cu microparticles to achieve a high‐resolution ML platform. Our approach effectively addressed the inherent spectral broadness of ZnS:Cu, which has been a major limitation in ML‐based sensing applications. By leveraging the high absorption of F8BT in the blue region below 490 nm, we demonstrated that selective spectral filtration can significantly enhance signal clarity and spectral resolution. We confirmed that the F8BT shell efficiently narrows the ML emission spectrum, reducing the FWHM to 55 nm, compared to 94 nm for uncoated ZnS:Cu. This spectral refinement was further validated by CIE color coordinate analysis, which showed a distinct shift toward pure green emission, eliminating unwanted blue spectral components. Furthermore, we observed that the ZnS:Cu@F8BT platform exhibited the least intensity loss, despite its high absorption capability. This unexpected result was attributed to an ML photon recycling effect, where the absorbed ML photons from ZnS:Cu were re‐emitted by F8BT, effectively compensating for the expected intensity loss. To demonstrate the practical applicability of this high‐resolution ML platform, we implemented a press‐sensitive color tracking system using ZnS:Cu@F8BT. The system successfully distinguished between blue and green ML signals, highlighting the superior spectral resolution enabled by our chromatic filtration strategy. The signal differentiation capability was further confirmed through real‐time intensity measurements, where the ZnS:Cu@F8BT system effectively eliminated spectral overlap that was present in conventional ZnS:Cu‐based sensors. Taken collectively, our findings establish a scalable material design framework that integrates chromatic filtration and ML signal compensation, enabling high‐resolution, multi‐signal processing with minimal spectral interference for advanced stress‐sensing and interactive device applications.

## 4 Experimental Section

### Chemicals and Reagents

Blue–Green ML phosphor (ZnS:Cu, 25 ± 5 µm) was purchased from KPT cooperation. Polydimethylsiloxane and curing agent (PDMS, Sylgard 184 product) were purchased from the Wacker corporation. 9,9‐Dioctylfluorene‐2,7‐diboronic acid bis(1,3‐propanediol) ester, tetrakis(triphenylphosphine)‐palladium(0), and 2,1,3‐benzothiadiazole were purchased from Sigma–Aldrich. 4,7‐Dibromo‐2,1,3‐benzothiadiazole^[^ ^26^ ^]^ was synthesized using previously published method.

### Synthesis of Poly(9,9‐Dioctylfluorene‐Altbenzothiadiazole) (F8BT)

9,9‐Dioctylfluorene‐2,7‐diboronic acid bis (1,3‐propanediol) ester (0.5 g, 1 mmol) and 4,7‐dibromo‐2,1,3‐ benzothiadiazole (0.29 g, 1 mmol) were added in a three necked round‐bottom flask. Then, dry toluene (14 mL) and 2 m K_2_CO_3_ aqueous solution (8 mL) were added into the reaction flask under argon. After the addition of (PPh_3_)_4_Pd(0) (81 mg, 0.07 mmol) and five drops of Aliquat 336 as a phase‐transfer catalyst, the mixture was then stirred at 90 °C for 36 h. After the reaction, the reaction mixture was cooled to room‐temperature and was added slowly to methanol. The resulting precipitated powder was isolated by filtration and washed with water, methanol, and acetone repeatedly, for removal of catalyst and salt. The polymer was dissolved in chloroform and precipitated in methanol/acetone twice to decrease in polydispersity by removing small molecules with oligomers. Finally, a yellow powder was obtained after drying under vacuum. Yield: 68% (0.30 g). ^1^H NMR (300 MHz, CDCl_3_):d = 8.14− 8.07 (br, 4H), 8.02–7.98 (br, 4H), 2.25–2.15 (br, 4H), 1.29–1.14 (br, 24H), 1.05–0.94 (br, 4H), 0.86–0.83 (br, 6H) ppm. 13CNMR (CDCl_3_):d = 154.43, 151.81, 140.93, 136.51, 133.67, 128.35, 128.00, 124.07, 120.07, 55.48, 40.25, 31.86, 30.14, 29.29, 24.09, 22.63, 14.07 ppm. Anal. Calcd for C_31_H_34_N_2_S (%): C 80.41; H 8.11; N 5.36; S 6.13. Found: C 78.55; H 7.39; N 5.85; S 6.58. Mn = 31.7k g mol^−1^, Mw = 47,4k g mol^−1^, PDI = 1.49

### Preparation of ZnS:Cu@F8BT Core–Shell Structure

The ZnS:Cu@F8BT core–shell structure was synthesized using a dip‐coating process based on physical adsorption principles. Initially, 1 mg of F8BT was dissolved in 0.5 mL of chloroform in a 20 mL vial to prepare the coating solution. Subsequently, 9.5 mL of hexane was added to this solution as a poor solvent for F8BT, facilitating adsorption. After adding 6 g of ZnS:Cu phosphor to the vial, the mixture was agitated to ensure uniform coating. The coated phosphor was washed twice with a mixed solvent (chloroform:hexane = 0.5:9.5, v/v) to remove any unabsorbed F8BT. Finally, a single wash with pure hexane was conducted. The coated ZnS:Cu@F8BT powder was then dried in an oven at 100 °C to remove any residual solvents.

### Fabrication of Mechanoluminescent Composite

The prepared ZnS:Cu@F8BT powder was incorporated into a PDMS matrix. The PDMS precursor and curing agent were mixed at a 10:1 weight ratio. The ZnS:Cu@F8BT powder was then blended with the PDMS mixture at a 7:3 weight ratio (ZnS:Cu@F8BT). This composite mixture was cast into a stainless‐steel mold and left undisturbed under ambient conditions for ≈20 min to allow self‐degassing and removal of trapped air bubbles. The mixture was then thermally cured in an oven at 100 °C for 1 h to obtain the final ML composite.

### Characterization

Photoluminescence (PL) spectra were measured using a Fluorescence Spectrophotometer RF‐6000 (Shimadzu). Mechanoluminescence (ML) spectra were obtained using a CS‐2000 spectroradiometer (Konica Minolta). To evaluate the ML optical properties, a custom‐built stretching/releasing setup was employed, applying a consistent frequency of 600 rpm to the ML film. Absorption spectra were recorded using a UV‐2600 UV–vis spectrophotometer (Shimadzu). Transmission electron microscopy (TEM) images of the core–shell structures were acquired using a JEM 2100F (JEOL). High‐resolution scanning electron microscopy (HR‐SEM) and energy‐dispersive X‐ray spectroscopy (EDS) mapping were performed with an HR‐SEM Sigma 300 (Carl Zeiss). Time‐resolved photoluminescence (TRPL) measurements were conducted using a FluoroMax Plus spectrofluorometer equipped with a time‐correlated single‐photon counting (TCSPC) system (HORIBA). The fluorescence images of ZnS:Cu@F8BT embedded in PDMS were obtained using IX73P2F fluorescence microscope (Olympus) at the micro‐scale. Gel permeation chromatography (GPC) was used for determination of molecular weights against polystyrene standards (From 1.32k to 791k g mol^−1^). Tetrahydrofuran (THF) was used as the eluent, and GMHHRM columns and a Bischoff LAMBDA 1000 detector were employed. Hyperspectral Photoluminescence Microscopy was conducted using the PI‐MAX4:1024B/EM system. A 405 nm laser was directed onto the back‐focal plane of the 100x objective, producing a near‐uniform illumination profile of ≈150 µm in diameter on the sample. The spatial resolution of the Photon Etc IMA microscope was limited by diffraction, ≈300 nm in this case. To detect only specific wavelengths, a spectral volume Bragg grating was placed before the camera, providing a spectral resolution of ≈2.5 nm. All images were captured using an iPhone 14 Pro (Apple Inc.) equipped with a 48 MP main camera (1/1.28‐inch sensor size, f/1.78 aperture) at the highest resolution setting (48 MP ProRAW mode). Exposure and ISO were manually adjusted to prevent over‐saturation of the bright ML signals while maintaining background darkness.

### Statistical Analysis

Quantitative data with error bars are presented as mean ± standard deviation (SD). Sample sizes (n) for each experiment and error bars are specified in the figure legends. Statistical significance was assessed using paired *t*‐tests where applicable. All analyses were performed using OriginPro 2023.

## Conflict of Interest

The authors declare no conflict of interest.

## Author Contributions

H.I.J. and S.E.C. equally contributed to this work. H.I.J and S.E.C. conceptualized this work. S.E.C., E.P. and H.I.J. characterized all ML samples and analyzed PL and ML. S.E.C., H.I.J. and S.B. analyzed the overall data in this study and wrote the manuscript. N.W.K. contributed to the UV–vis spectroscopy and PLE measurement. H.I.J. and X.W.C contributed hyperspectral PL microscopy analysis. H.L. and J.K. synthesized F8BT conjugated polymer and analyze absorption and PL of F8BT sample. H.I.J., S.E.C. and S.B. contributed to the preparation of the manuscript and provided insights for organizing this study. S.D.S and D‐W.K. contributed to the preparation of the manuscript and provided insights for organizing this study. S.D.S, J.K., S.B. and H.C. supervised this work. All the authors contributed their knowledge to the discussion and validated the manuscript.

## Supporting information



Supporting Information

## Data Availability

The data that support the findings of this study are available from the corresponding author upon reasonable request.
