# Supplementary material for: High‐Resolution Mechanoluminescent Haptic Sensor via Dual‐Functional Chromatic Filtration by a Conjugated Polymer Shell
Source: Adv Mater. 2025 Aug 14;37(44):e08917. doi: 10.1002/adma.202508917 (PMC12592900; doi:10.1002/adma.202508917)
Supplement: Supplementary file 1 — Supporting Information [file ADMA-37-e08917-s001.docx]

Supporting Information

**High-resolution mechanoluminescent haptic sensor via dual-functional chromatic filtration by a conjugated polymer shell**

*Hong In Jeong, So Eun Choi, Xian Wei Chua, Nam Woo Kim, Eleni Pyrilli, Hyosun Lee, Dong-Won Kang, Bo Ram Lee, Samuel D. Stranks, Jongho Kim*, Sujoy Bandyopadhyay*, Hyosung Choi**

Dr. H. I. Jeong, X.W. Chua, E. Pyrilli, Prof. Dr. S. D. Stranks

Department of Chemical Engineering and Biotechnology, University of Cambridge, Cambridge, CB3 0AS, United Kingdom

Prof. Dr. D.-W. Kang

School of Energy Systems Engineering, Chung-Ang-University, Seoul 04763, Republic of Korea

Prof. Dr. B.R. Lee

School of Advanced Materials Science and Engineering, Sungkyunkwan University, Suwon, 16419, Republic of Korea

H. Lee, Prof. Dr. J. Kim

Department of Textile System Engineering, Kyungpook National University, Daegu 41566, Republic of Korea

E-mail: sci.jongho@knu.ac.kr

S.E. Choi, N.W. Kim, Dr. S. Bandyopadhyay*,* Prof. Dr. H. Choi

Department of Chemistry, Research Institute for Natural Sciences, and Research Institute for Convergence of Basic Science, Hanyang University, Seoul 04763, Republic of Korea

E-mail: [hschoi202@hanyang.ac.kr](mailto:hschoi202@hanyang.ac.kr)


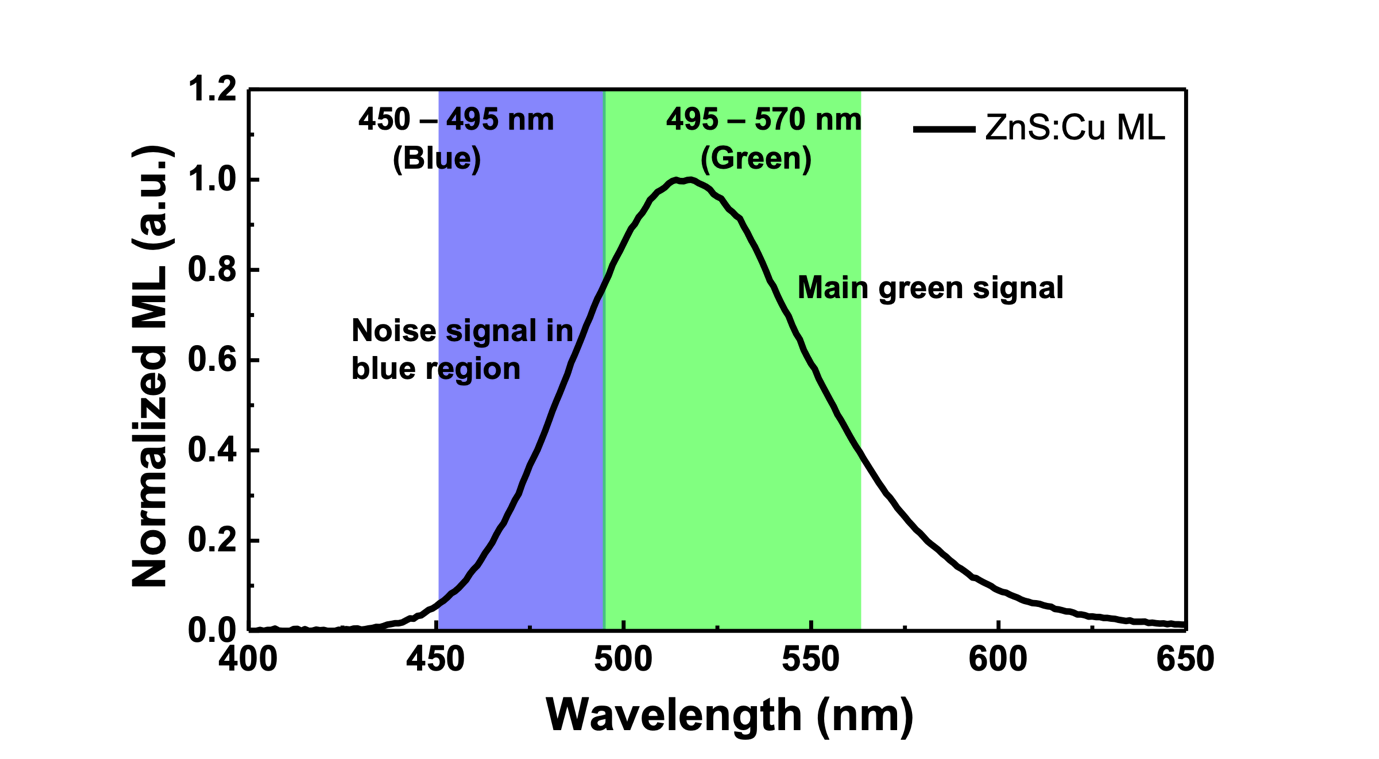


**Figure S1.** ML spectrum of typical ML platform with ZnS:Cu microparticles indicating the colour region of blue and green.

**
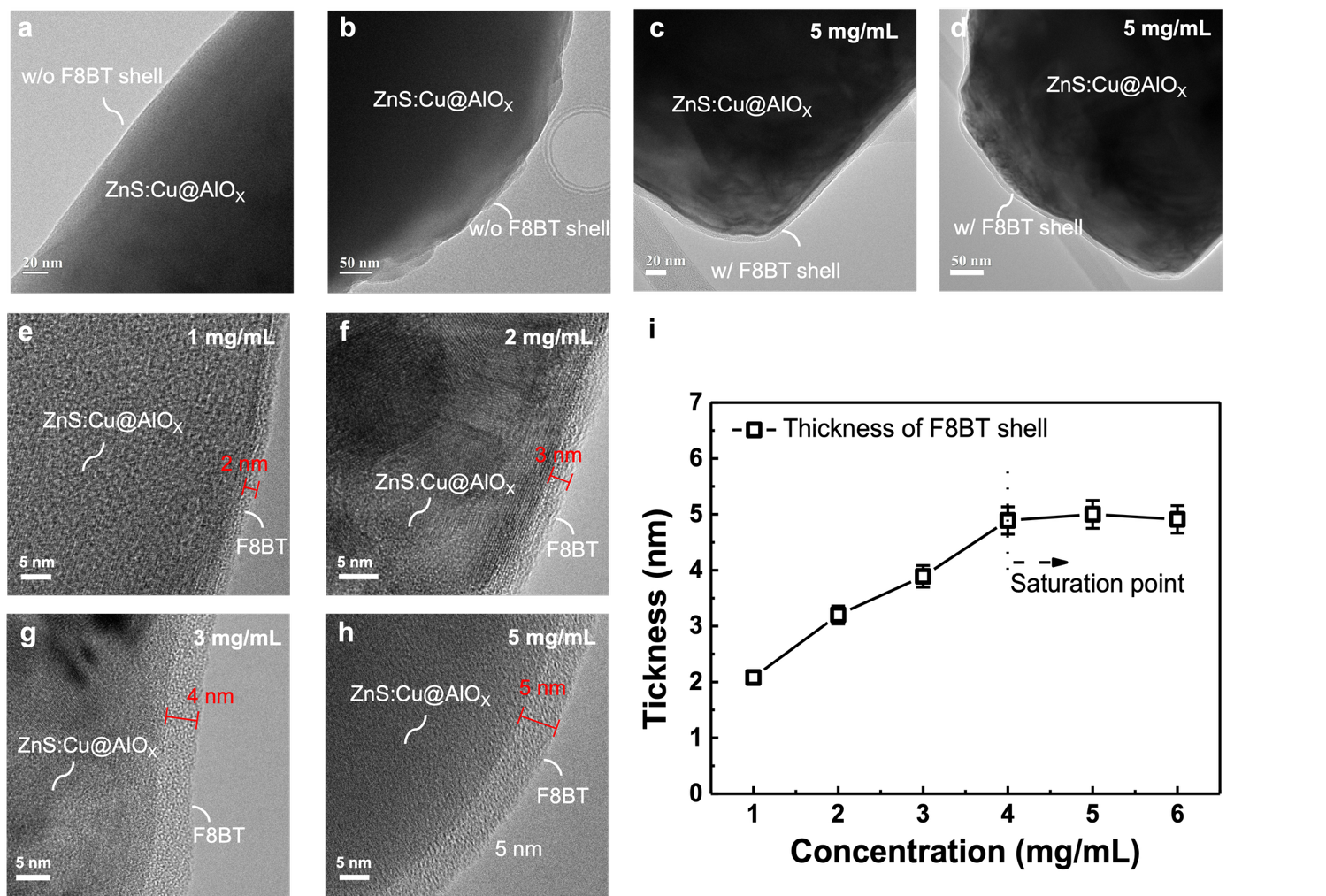
**

**Figure S2.** TEM images of ZnS:Cu microparticle (a, b) with and (c, d) without an F8BT shell (prepared using an F8BT concentration of 5 mg/mL). TEM images showing the variation in F8BT shell thickness depending on the F8BT concentration of (e) 1 mg/mL**,** (f) 2 mg/mL**,** (g) 3 mg/mL and (h) 5 mg/mL**.** (i) Plot of F8BT shell thickness as a function of F8BT concentration (n = 3, error bars represent ±5%).


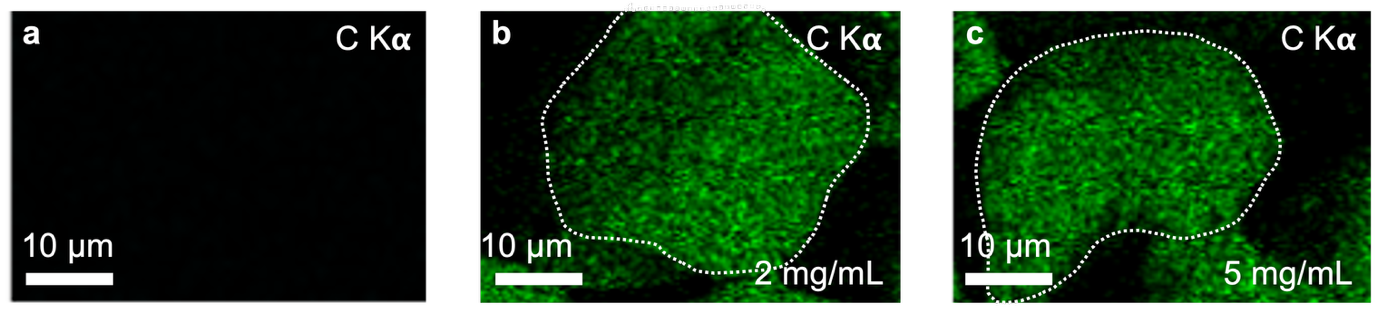


**Figure S3.** SEM/EDX results of ZnS:Cu microparticles depending on the F8BT concentration of (a) 0 mg/mL, (b) 2 mg/mL, (c) 5 mg/mL.

**Table S1.** SEM/EDX mapping profiles with weight percentage (wt%) and atomic percentage (at%) of ZnS:Cu microparticles w/o and w/ coating process of F8BT shell.

|  | **w/o F8BT shell** | | **w/ F8BT shell**  **(2 mg/mL)** | | **w/ F8BT shell**  **(5 mg/mL)** | |
| --- | --- | --- | --- | --- | --- | --- |
| **Element** | **wt%** | **at%** | **wt%** | **at%** | **wt%** | **at%** |
| **C** | 0.00 | 0.00 | 10.21 | 22.31 | 26.16 | 42.32 |
| **O** | 6.72 | 14.71 | 18.74 | 30.74 | 20.73 | 25.18 |
| **Al** | 39.93 | 51.85 | 20.81 | 20.25 | 14.81 | 10.67 |
| **S** | 8.70 | 9.50 | 15.58 | 12.76 | 9.74 | 5.91 |
| **Cu** | 0.45 | 0.25 | 2.70 | 1.11 | 0.30 | 9.46 |
| **Zn** | 44.21 | 23.69 | 31.96 | 12.83 | 21.43 | 6.46 |

**
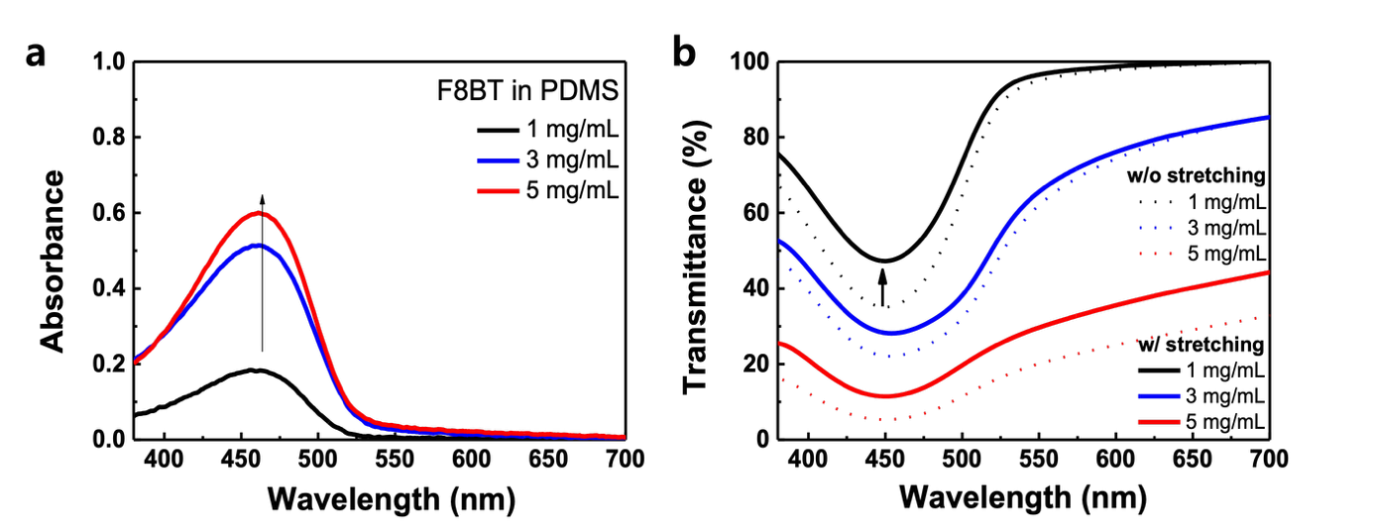
**

**Figure S4.** (a) Absorbance and (b) transmittance spectrum of the F8BT-PDMS film depending on the F8BT concentration.


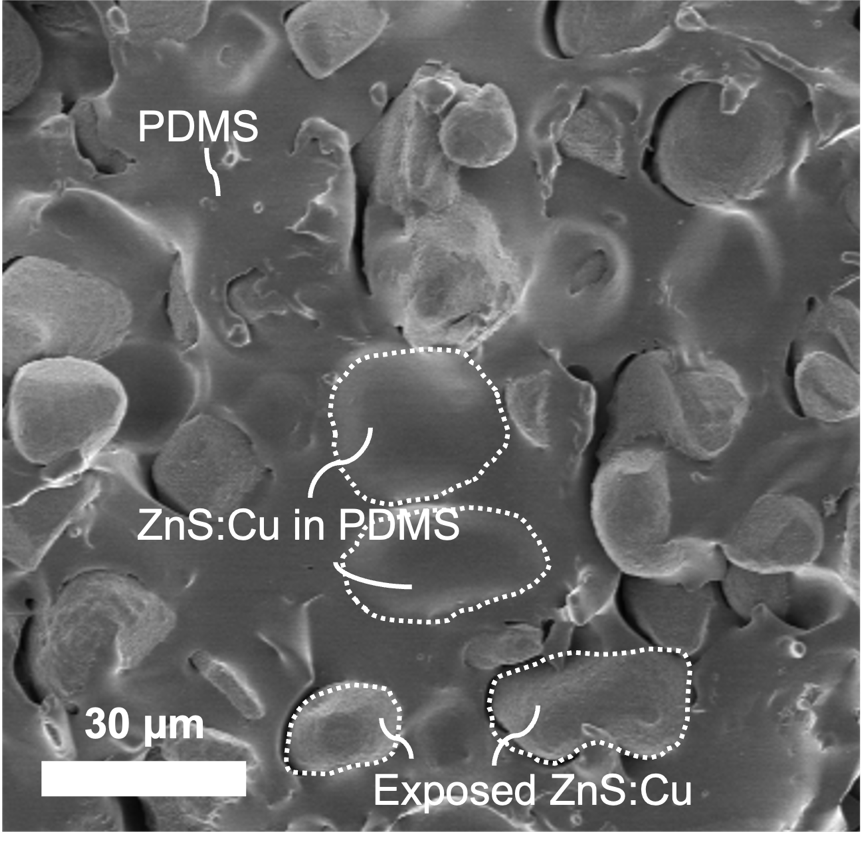


**Figure S5.** SEM image for the surface of mixed sample (ZnS:Cu + F8BT-PDMS matrix).


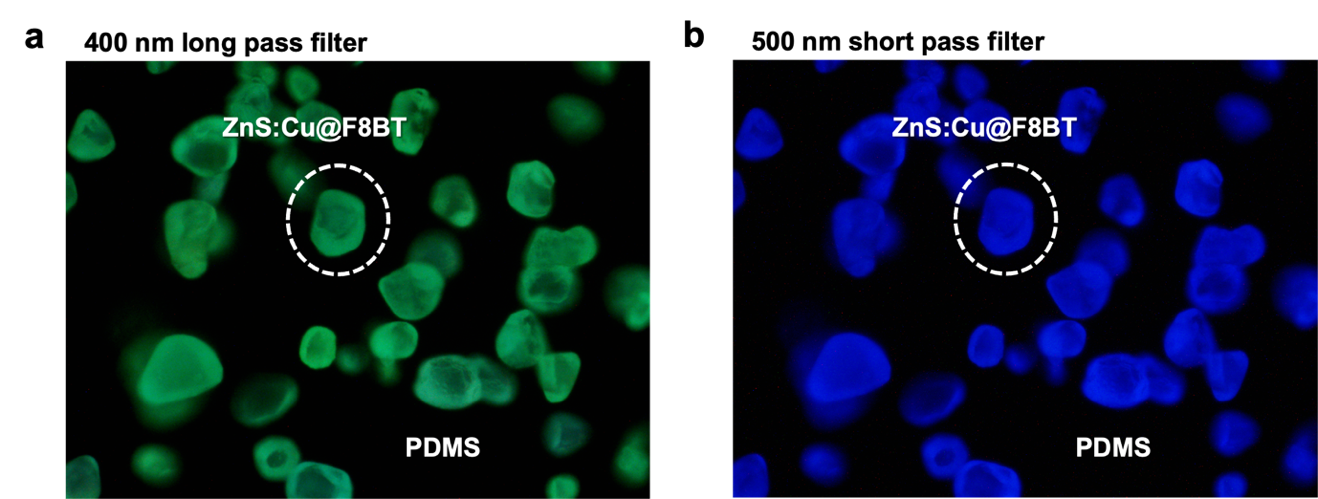


**Figure S6.** Fluorescence microscopy images of ZnS:Cu@F8BT particle in PDMS by using of (a) 400 nm long pass filter and (b) 500 nm short pass filter.


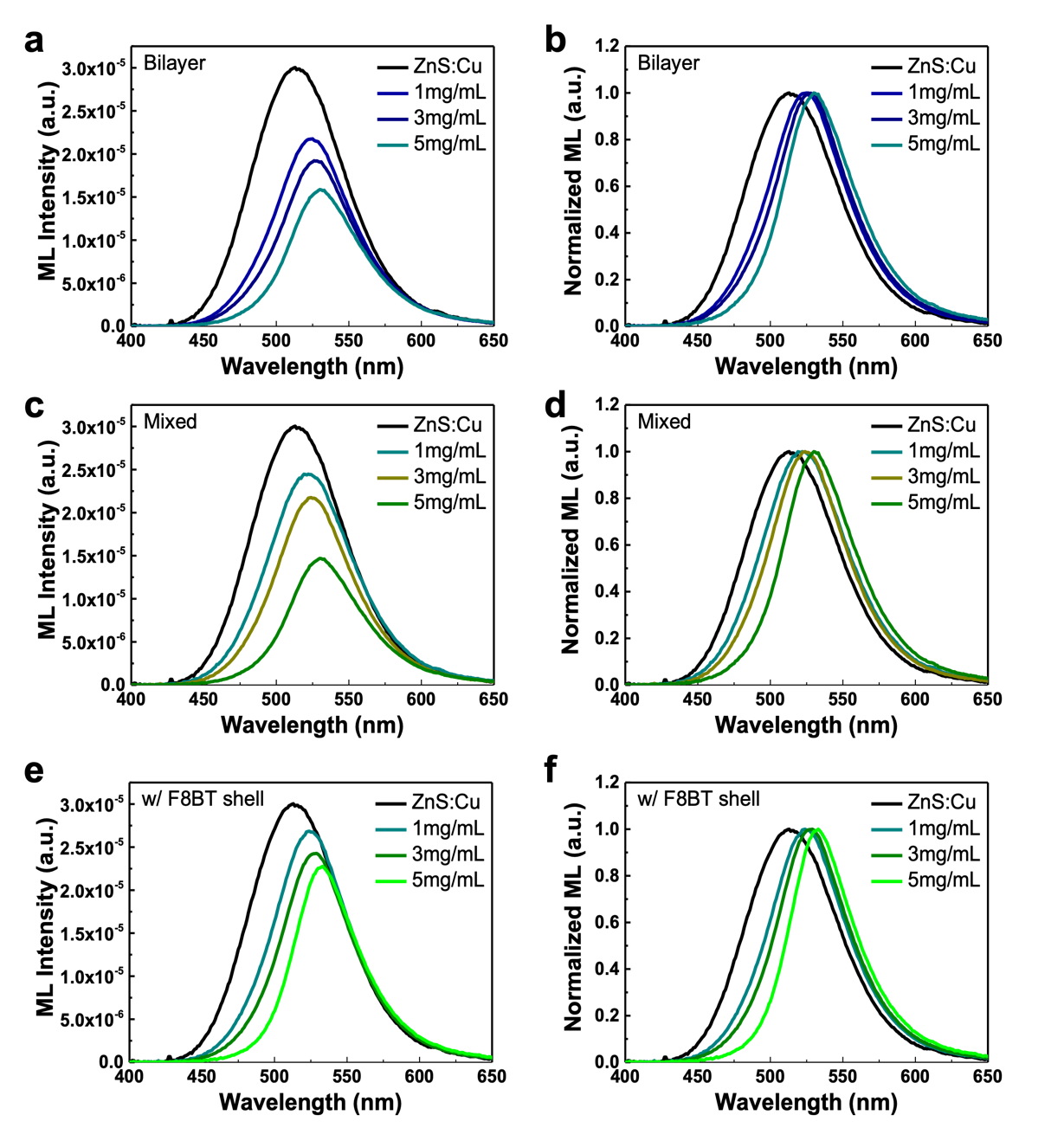


**Figure S7.** ML spectra of (a, b) bilayer-, (c, d) mixed-, and (e, f) ZnS:Cu@F8BT (w/ F8BT shell) samples at F8BT concentrations of 1 mg/mL, 3 mg/mL, and 5 mg/mL, respectively.

**
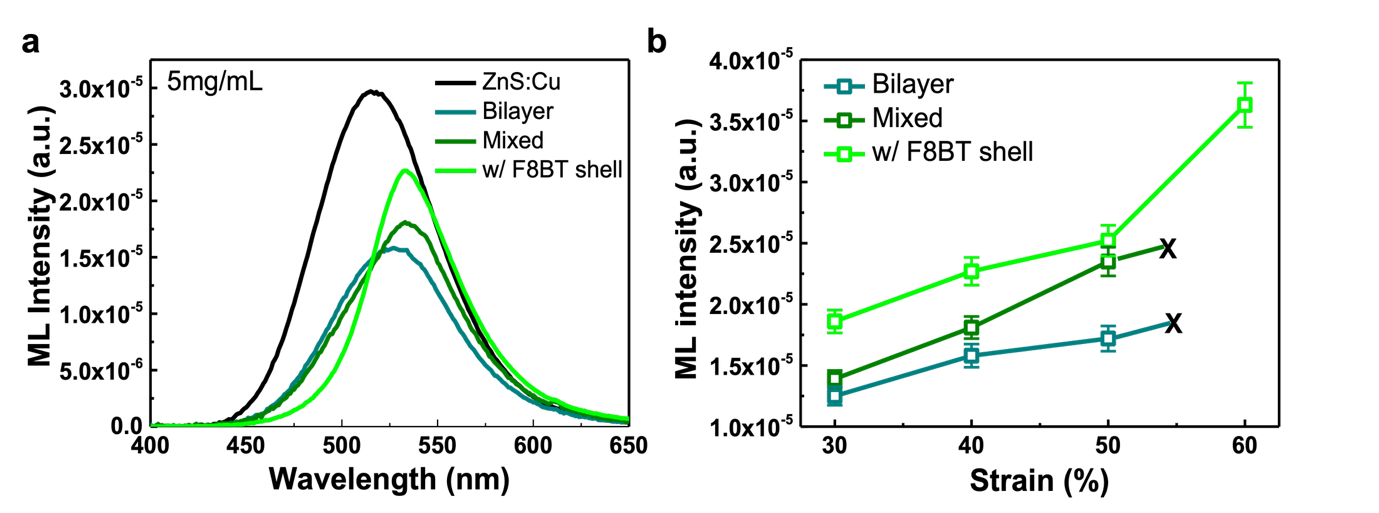
**

**Figure S8.** (a) Relative ML spectra of each sample (bilayer, mixed, and ZnS:Cu@F8BT shell) at an F8BT concentration of 5 mg/mL under 40% strain. (b) ML intensity of each sample (bilayer, mixed, and ZnS:Cu@F8BT shell) at an F8BT concentration of 5 mg/mL as a function of tensile strain ranging from 30% to 60% (n = 5, error bars represent ±6%).


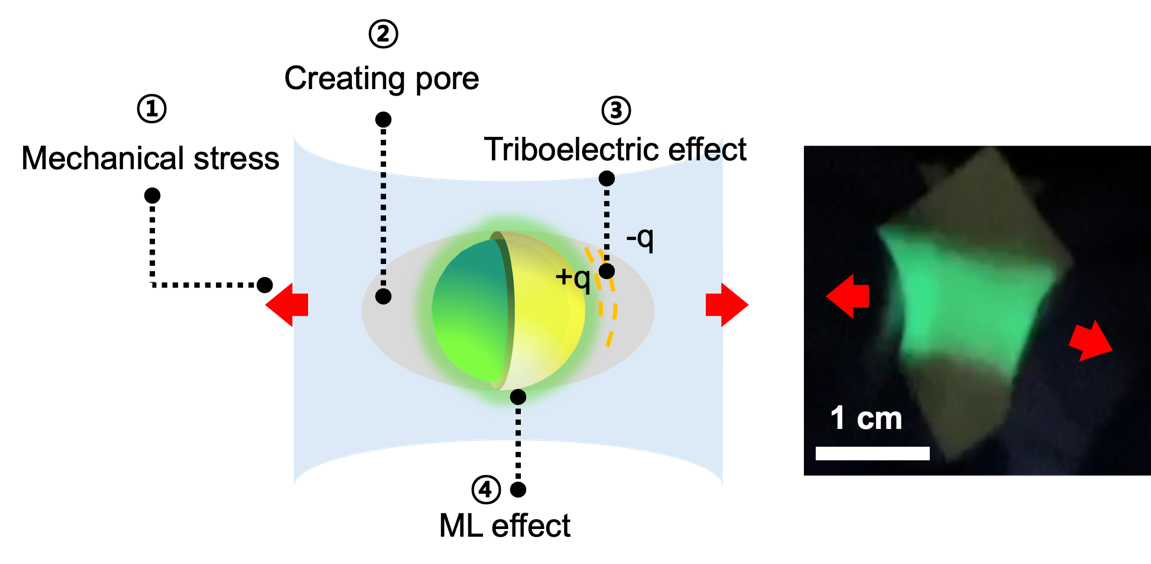


**Figure S9.** General ML mechanism attributed to the triboelectric effect. Inset image indicates the ML effect by the stitching motion by human fingers.

**Note S1**

Fundamentally, the ML mechanism is well known to be driven by the triboelectric effect as following steps. ① An external mechanical stimulus such as stretching motion is applied. ② The induced physical deformation creates interfacial pore between the luminescent material and the polymer matrix. ③ Within these pores, interfacial charge separation leads to the formation of a triboelectric field. ④ The generated triboelectric field induces structural deformation in ZnS:Cu, thereby activating the ML effect.

**
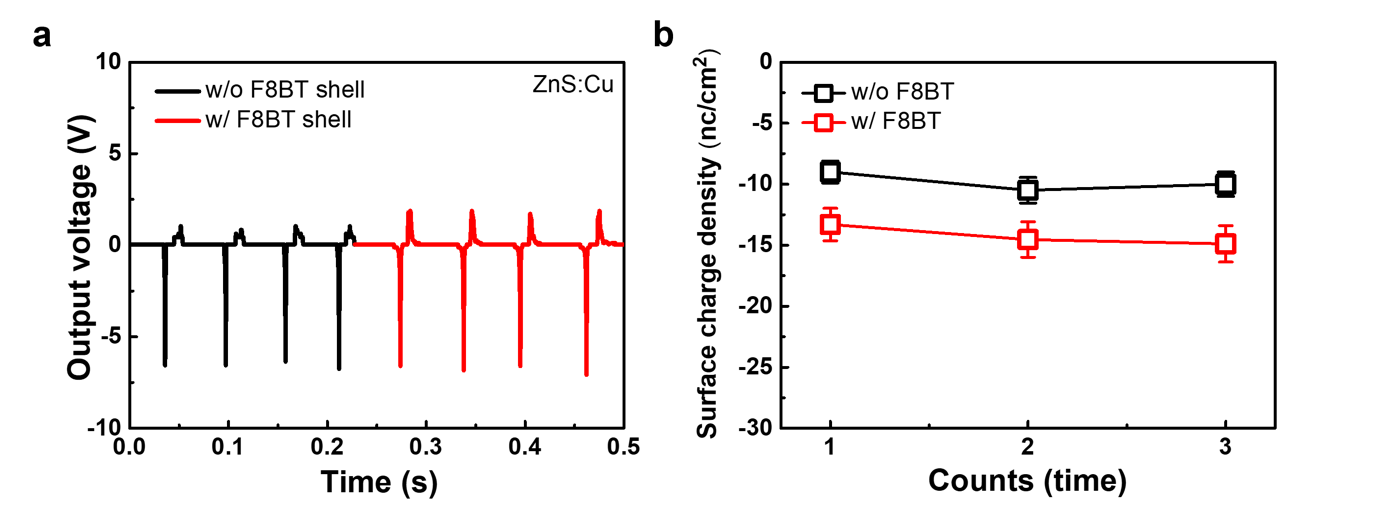
**

**Figure S10.** (a) Output voltage and (b) surface charge density of the ZnS:Cu microparticles w/ and w/o F8BT shell (n = 5, error bars represent ±10%).


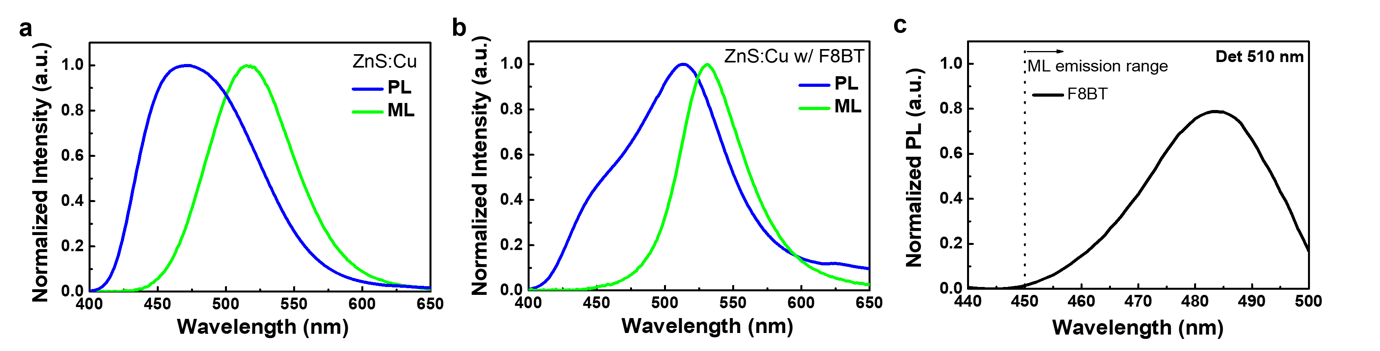


**Figure S11.** PL and ML spectra of (a) ZnS:Cu and (b) ZnS:Cu@F8BT. (c) PLE spectra of F8BT in PDMS matrix as a function of excitation energy ranging from 440 to 500 nm.


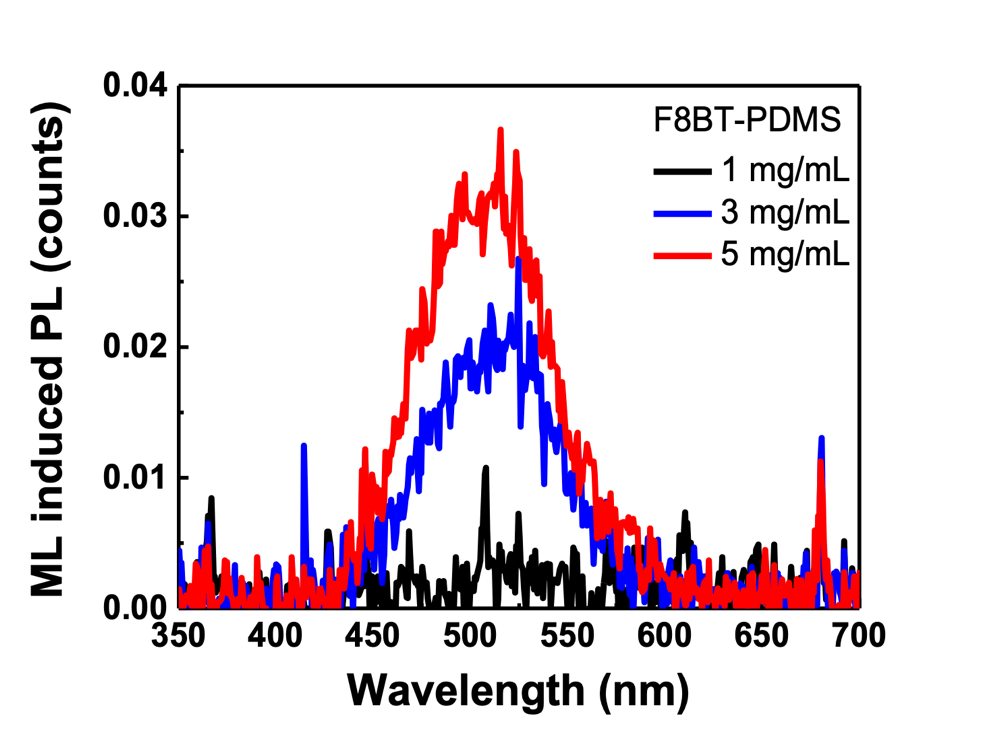


**Figure S12.** ML-induced secondary emission spectrum of the bi-layer sample depending on the F8BT concentration.

**
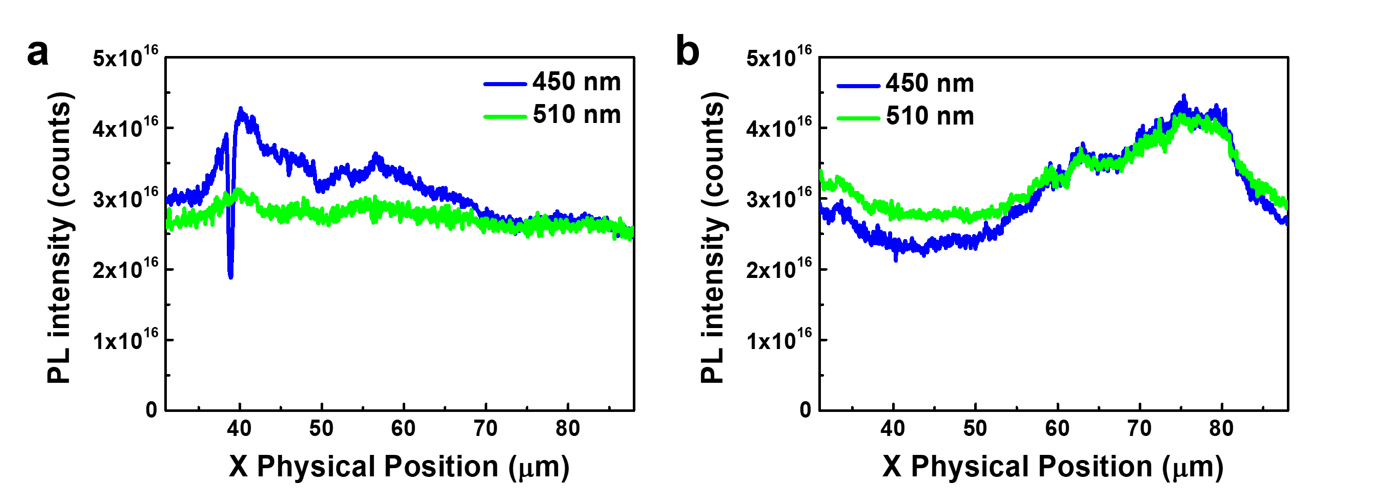
**

**Figure S13.** Line-scan profiles of PL intensity at 450 nm and 510 nm along the horizontal (x) axis across dots 1–5 in the PL maps shown in (a) Figure 3a and (b) Figure 3d.

**
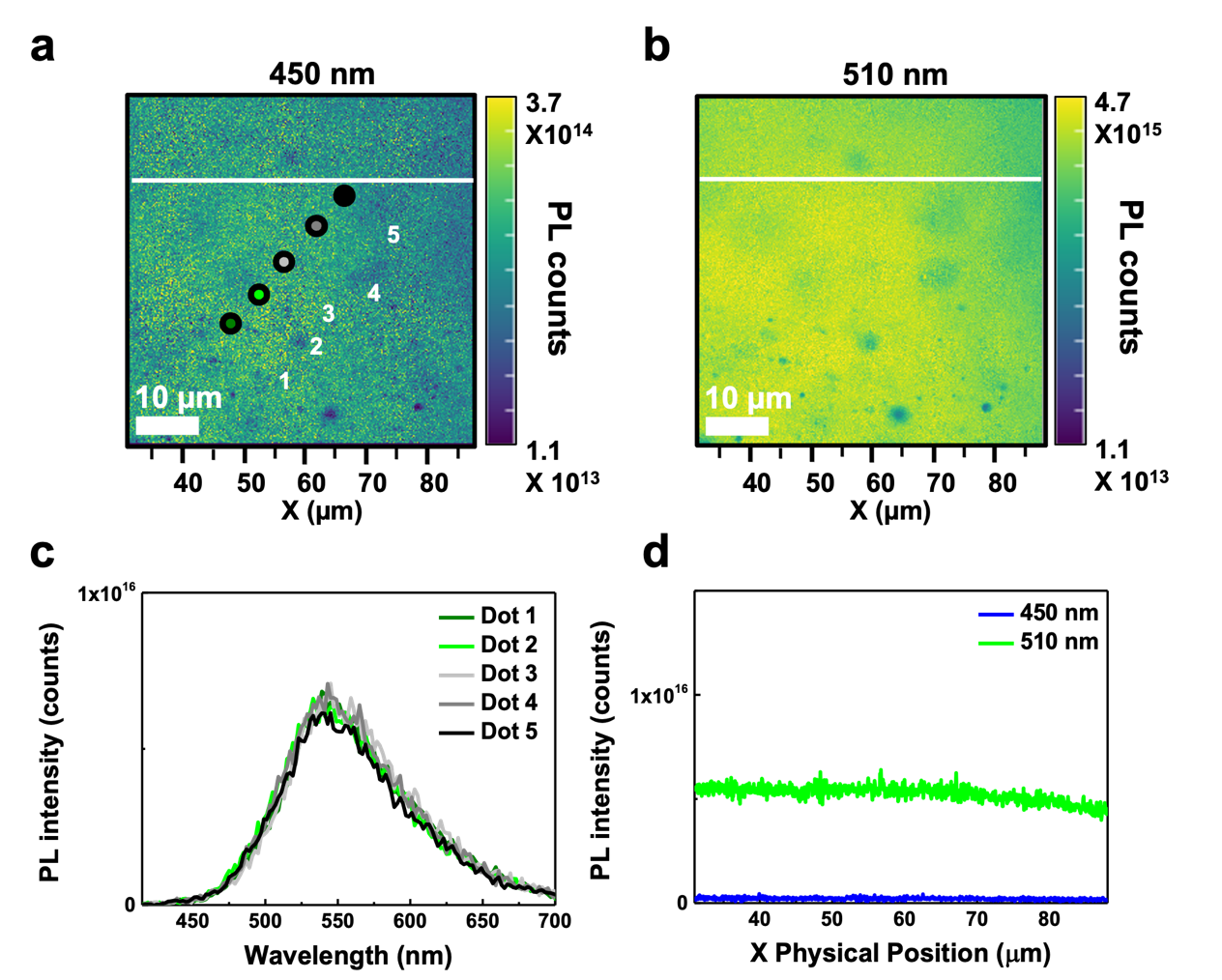
**

**Figure S14.** The top view of PL maps of bi-layer sample as a function of (a) 450 nm and (b) 510 nm. (c) PL spectra of each dot in (a). (d) Line-scan profiles of PL intensity at 450 nm and 510 nm along the horizontal (x) axis in the PL map of (a) and (b).

**Table S2.** Time decay values of ZnS:Cu@F8BT shell depending on the F8BT concentration at 450 nm and 510 nm emission component.

| **Em** |  | **τ_1_** | **τ_2_** | **τ_3_** | τ_avg_ | CHISQ |
| --- | --- | --- | --- | --- | --- | --- |
| **450 nm** | **Control** | 1.31 ms | 7.25 ms | 0.23 ms | 3.44 ms | 1.380 |
|  | **Low** | 1.39 ms | 7.51 ms | 0.23 ms | 3.52 ms | 1.280 |
|  | **High** | 1.44 ms | 7.81 ms | 0.24 ms | 3.61 ms | 1.355 |
| **510 nm** | **Control** | 3.03 ms | 15.1 ms | 0.62 ms | 6.32 ms | 1.326 |
|  | **Low** | 2.91 ms | 14.8 ms | 0.59 ms | 6.33 ms | 1.348 |
|  | **High** | 2.91 ms | 15.2 ms | 0.58 ms | 6.52 ms | 1.197 |


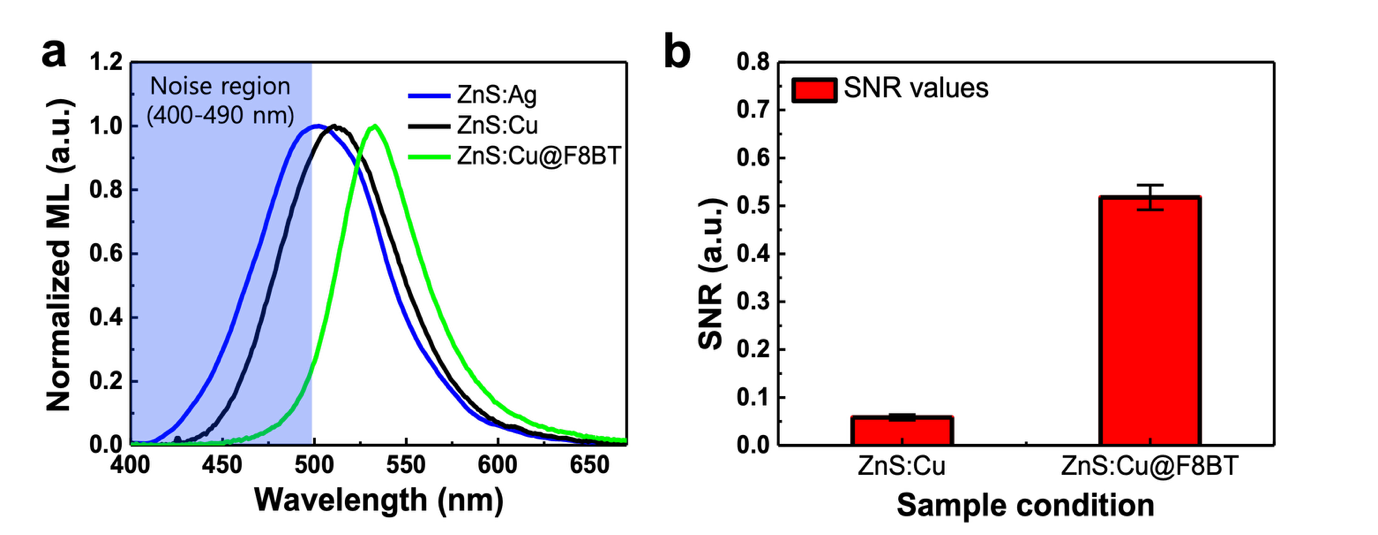


**Figure S15.** (a) ML spectra of ZnS:Ag and ZnS:Cu with and without the F8BT shell (concentration: 5 mg/mL) under the same stretching condition, showing the effect of the F8BT shell on spectral separation from the ZnS:Ag emission region. (b) SNR values for ZnS:Cu with and without the F8BT shell (n = 5, error bars represent ±5%), calculated as SNR = *I*_Green peak_/*I*_Blue region_, where *I*_Green peak_ is the maximum intensity of the green spectral region (500–550 nm) and *I*_Blue region_ is the integrated intensity of the overlapping blue region (400–490 nm).

**Table S3.** CIE color coordinates corresponding to S1 zone, S2 zone, and S2′ zone in Figure 4g, summarizing the chromaticity values of ZnS:Ag and ZnS:Cu with and without the F8BT shell.

| **Signal zone** | **Dot number** | **CIE coordinate** | |
| --- | --- | --- | --- |
|  |  | **x** | **y** |
| **S1 zone** | **1** | 0.192 | 0.469 |
|  | **2** | 0.195 | 0.477 |
|  | **3** | 0.199 | 0.485 |
|  | **4** | 0.201 | 0.495 |
|  | **5** | 0.203 | 0.501 |
|  | **6** | 0.209 | 0.511 |
|  | **7** | 0.211 | 0.521 |
| **S2 zone** | **1** | 0.205 | 0.521 |
|  | **2** | 0.209 | 0.550 |
|  | **3** | 0.212 | 0.559 |
|  | **4** | 0.219 | 0.562 |
|  | **5** | 0.225 | 0.570 |
|  | **6** | 0.231 | 0.573 |
| **S2’ zone** | **1** | 0.299 | 0.625 |
|  | **2** | 0.295 | 0.629 |
|  | **3** | 0.291 | 0.631 |
|  | **4** | 0.286 | 0.641 |
|  | **5** | 0.283 | 0.650 |
